# Intracellular Invasion of *Orientia tsutsugamushi* Activates Inflammasome in ASC-Dependent Manner

**DOI:** 10.1371/journal.pone.0039042

**Published:** 2012-06-18

**Authors:** Jung-Eun Koo, Hye-Jin Hong, Andrea Dearth, Koichi S. Kobayashi, Young-Sang Koh

**Affiliations:** 1 Department of Microbiology and Immunology, Brain Korea 21 Program, Jeju National University School of Medicine, Jeju, Jeju-Do, South Korea; 2 Institute of Medical Science, Jeju National University, Jeju, Jeju-Do, South Korea; 3 Department of Cancer Immunology and AIDS, Dana-Farber Cancer Institute, Boston, Massachusetts, United States of America; 4 Department of Microbiology and Immunobiology, Harvard Medical School, Boston, Massachusetts, United States of America; Louisiana State University, United States of America

## Abstract

*Orientia tsutsugamushi*, a causative agent of scrub typhus, is an obligate intracellular bacterium, which escapes from the endo/phagosome and replicates in the host cytoplasm. *O. tsutsugamushi* infection induces production of pro-inflammatory mediators including interleukin-1β (IL-1β), which is secreted mainly from macrophages upon cytosolic stimuli by activating cysteine protease caspase-1 within a complex called the inflammasome, and is a key player in initiating and maintaining the inflammatory response. However, the mechanism for IL-1β maturation upon *O. tsutsugamushi* infection has not been identified. In this study, we show that IL-1 receptor signaling is required for efficient host protection from *O. tsutsugamushi* infection. Live *Orientia*, but not heat- or UV-inactivated *Orientia*, activates the inflammasome through active bacterial uptake and endo/phagosomal maturation. Furthermore, *Orientia*-stimulated secretion of IL-1β and activation of caspase-1 are ASC- and caspase-1- dependent since IL-1β production was impaired in *Asc*- and caspase-1-deficient macrophages but not in *Nlrp3*-, *Nlrc4*- and *Aim2*-deficient macrophages. Therefore, live *O. tsutsugamushi* triggers ASC inflammasome activation leading to IL-1β production, which is a critical innate immune response for effective host defense.

## Introduction

*Orientia tsutsugamushi*, an obligate intracellular bacterium which is transmitted by the bite of the larvae of certain trombiculid mites, is the causative agent of scrub typhus (tsutsugamushi disease). Scrub typhus is characterized by fever, rash, eschar, pneumonitis, meningitis, and disseminated intravascular coagulation and often becomes fatal due to severe multiple organ failure without appropriate treatment [1], [2]. *O. tsutsugamushi* usually infects endothelial cells, macrophages, polymorphonuclear leukocytes (PMN), and lymphocytes in patients or in animal models [3]–[7]. Proinflammatory cytokines, such as TNF-α, IL-1β and interleukin-6 (IL-6), increase markedly in patients with scrub typhus, and attribute to the high fever occurring in most scrub typhus patients [8]. Such host responses against *O. tsutsugamushi* may involve the activation of specialized pattern recognition receptors (PRR) in the cells, leading to the production of proinflammatory mediators.

The innate immune system provides the first line of protection against pathogens. Major functions of the innate immune system include recruiting immune cells to sites of infection and the activation of the complement cascade and the adaptive immune system. Host immune cells sense microbial infection using pattern recognition receptors (PRRs) that recognize molecular signatures known as pathogen-associated molecular patterns (PAMPs) [9]. PRRs include Toll-like receptors (TLRs), NLR or nucleotide binding domain (NBD), leucine rich repeat (LRR) family of proteins [10], [11] and retinoid acid-inducible gene I (RIG-I)-like receptors (RLRs) and contribute to immune activation in response to diverse stimuli, including infection or tissue injury [12]. These PRRs are expressed either on the cell membrane or in endosomal compartments or the cytoplasm.

Recent studies have shown the existence of a cytosolic detection system for intracellular PAMPs. These intracellular PAMPs are also recognized by a PRR family of cytosolic NLRs. NLRs consist of three domains characterized by an amino-terminal protein interaction domain, a central nucleotide-binding domain and a carboxy-terminal LRR (leucine-rich repeat) domain [13]. NLR proteins can be subclassified by their N-terminal protein interaction domains into CARD containing (NLRC), Pyrin containing (NLRP) or other NLR family proteins [11]. So far, at least 23 human and 34 murine NLR genes have been identified, although the physiological function of most NLRs remains poorly understood [14]. With the exception of Nod1 and Nod2, which are involved in the activation of inflammatory gene expression, several NLRs are involved in the activation of caspase-1-activating complexes called inflammasomes [15]. These NLRs, including Nlrp1, Nlrp3 and Nlrc4, respond to various PAMPs or damage associated molecular patterns and lead to the release of the IL-1 family of inflammatory cytokines including IL-1β, IL-18 and IL-33 through the formation of the inflammasome [16]. Nlrp1 senses the *Bacillus anthracis* lethal toxin, which is delivered into the cytoplasm by receptor-mediated endocytosis [17]. Nlrc4 senses bacterial flagellin and components of the type III secretion system (TSSS) such as PregJ-like protein through Naip5 and Naip2, respectively [18], [19]. Nlrp3 senses exogenous and host danger signals such as pore-forming toxins, extracellular ATP and crystals such as uric acid, cholestrol, silica, asbestos or alum [20]. Activation of the inflammasome also causes programmed cell death called pyroptosis, which contributes to the elimination of pathogen-infected cells [21]. The inflammasome consists of NLRs, caspase-1 and the adaptor protein apoptosis-associated speck-like protein containing a carboxy-terminal CARD (ASC). Caspase-1, also known as IL-1β-converting enzyme, mediates the processing of the pro-form of these cytokines into mature forms, which results in the secretion of bioactive cytokines. ASC bridges the interaction between NLRs and caspase-1 in the inflammasome complexes by mediating homotypic interactions with its amino-terminal pyrin domain and carboxy-terminal CARD [22]. ASC has a specific role in caspase-1 activation, because secretion of TNF-α and IL-6 is not affected by ASC deficiency [23]. Recently, the absent in melanoma 2 (AIM2) has been identified as a novel inflammasome component involved in the recognition of cytosolic DNA during viral and bacterial infection such as *Listeria monocytogenes* and *Francisella tularensis* [24]–[30]. AIM2 is a type I IFN-inducible cytosolic protein containing an amino-terminal pyrin domain and a domain of carboxy-terminal hematopoietic interferon-inducible nuclear antigens with 200 amino acid repeats (HIN200) [24]. The HIN domain promotes binding of DNA, whereas the pyrin domain associates with ASC and forms the caspase-1-activating inflammasome. Like other inflammasomes, AIM2 leads to the processing and release of mature IL-1β and IL-18 and host cell death called pyroptosis via the activation of caspase-1.

Although *O*. *tsutsugamushi* infection induces severe inflammation accompanied with the production of proinflammatory cytokines including IL-1β, the mechanism by which *O. tsutsugamushi* activates innate immune responses has not been elucidated. In this study, we demonstrate that IL-1 receptor signaling is critical for effective host defense during O. *tsutsugamushi* infection. Furthermore, we found that the uptake of live bacteria by macrophages is essential for *O. tsutsugamushi*-induced inflammasome activation, which results in the release of IL-1β in an ASC adaptor protein- and caspase-1-dependent manner.

## Results

### IL-1 Receptor Signaling is Required for Effective Host Defense Against *O. tsutsugamushi* (OT) Infection

**Figure 1:**
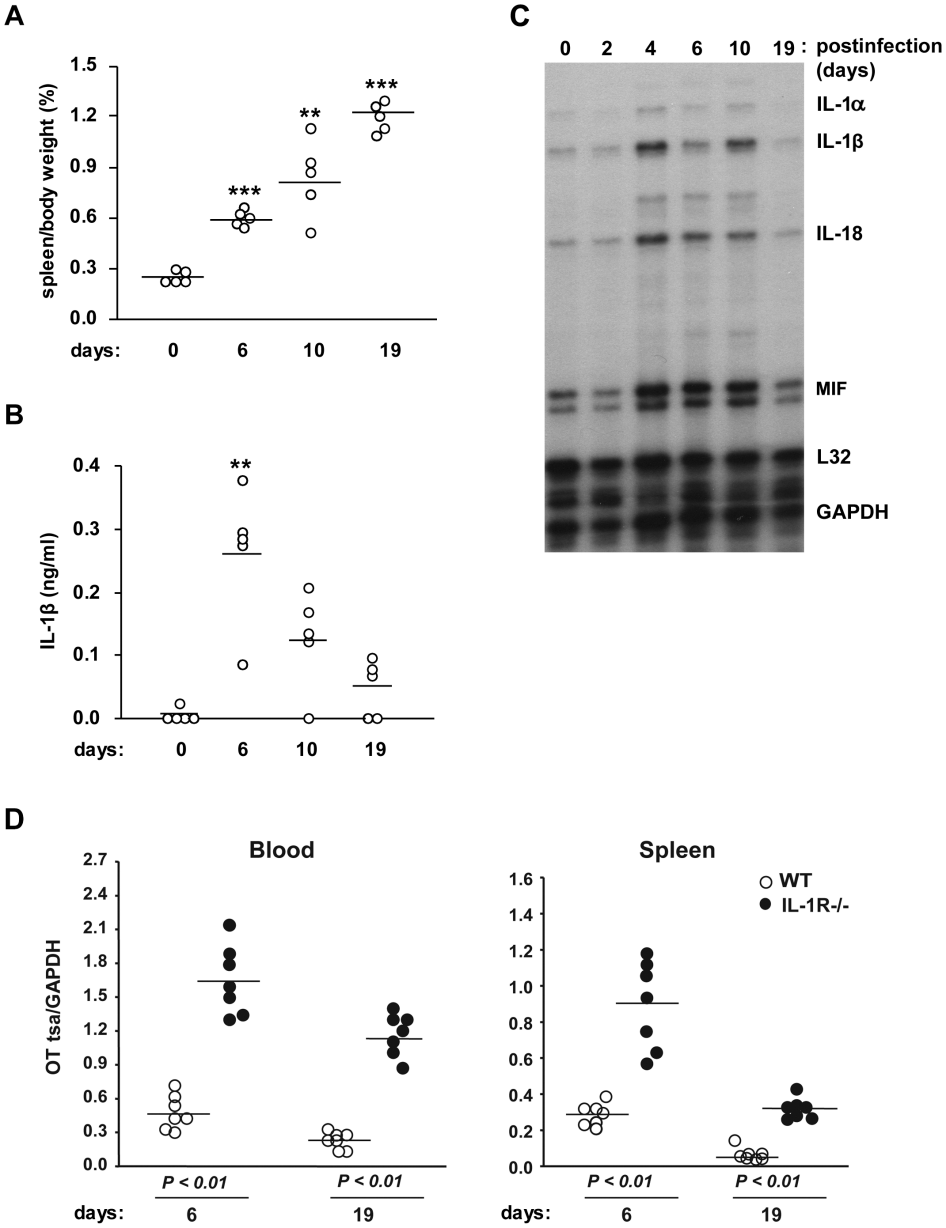
IL-1 receptor signaling is required for effective host protection from *O. tsutsugamushi* infection. (A) C57BL/6 mice (*n*=5) were *i.p.* inoculated with *O. tsutsugamushi* (OT) (5×10^6^ ICU) for the indicated time periods. Spleens were removed from infected mice and their weights were measured and normalized to body weight. (B) Serum IL-1β levels from infected mice were assessed by ELISA. A single circle represents an individual animal, and lines indicate the mean values. **p<0.01, ***p<0.001 versus uninfected mice. (C) Cytokine mRNA expression in the spleen from infected mice. At the indicated time periods after infection, total RNA was extracted and mRNA expression was determined by RNase protection assay. L32, a murine ribosomal protein; GAPDH, glyceraldehyde-3-phosphate dehydrogenase. (D) Age and sex-matched wild-type (WT, *n*=7) and IL-1R-deficient mice (*n*=7) were infected with *O. tsutsugamushi i.p.* for 6 and 19 days. *O. tsutsugamushi* loads in blood and spleen were quantified by qPCR using primers specific for the *O. tsutsugamushi tsa56* gene. Data were normalized by qPCR data for the GAPDH gene in host genomic DNA. A single circle represents an individual animal, and lines indicate the mean values. *p<0.01,* Wild-type vs. IL-1R−/−. Data are representative of three independent experiments in A-C.

To determine which proinflammatory cytokines play a critical role in host defense during OT infection, C57BL/6 mice were intraperitoneally infected with OT, and the mortality, splenomegaly and cytokine level in the serum were examined. The OT-infected mice did not show any clinical signs by day 6 postinfection. After this asymptomatic period, the mice became increasingly sick and were seriously ill by day 10 with increased amounts of ascites. The mice then gradually recovered afterwards and appeared normal by day 19. During the course of the disease, the mice developed splenomegaly that progressively became severe (Figure 1A). We found that OT infection induced IL-1β release in the serum, which peaked on day 6 (Figure 1B). The elevation of serum IL-1β levels seems partially due to increased transcripts of IL-1β, since mRNA levels of IL-1β and related cytokine IL-18 in the spleen assessed by RNA protection assay increased after OT infection, together with IL-1α and MIF, and peaked on day 4 (Figure 1C). This finding of IL-1β release during OT infection prompted us to examine the role of IL-1 receptor signaling in host defense against OT infection. We challenged wild-type and IL-1 receptor (IL-1R) deficient mice with OT and quantified bacterial numbers in the blood and spleen using qPCR. OT was detected in both blood and spleen in wild-type mice on day 6 and this bacterial load decreased by day 19 (Figure 1D). Strikingly, we found that IL-1R deficient mice were highly susceptible to OT, as demonstrated by the significantly elevated numbers of OT in both the blood and spleen, indicating that IL-1 receptor signaling is important for effective protection of hosts from OT infection.

### Live *O. tsutsugamushi* Infection Activates IL-1β Processing and Secretion

**Figure 2:**
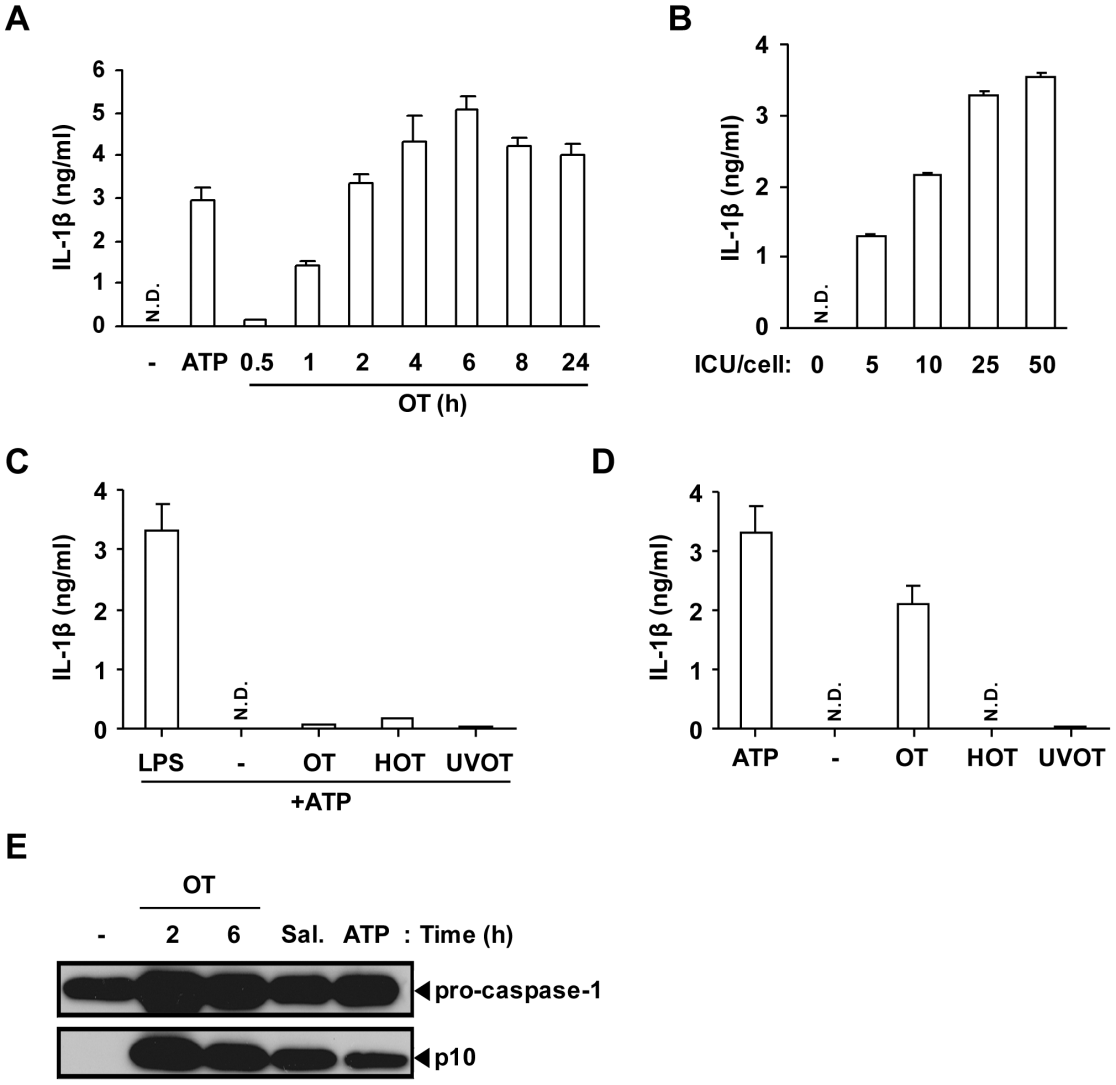
Live *O. tsutsugamushi* infection activates caspase-1 and induces IL-1β secretion in LPS-primed BMDMs. (A, B, D) BMDMs were primed with LPS (10 ng/ml) for 16 h and infected with OT. IL-1β release was assessed by ELISA. (A) LPS-primed BMDMs were treated with ATP (5 mM) for 3 h or infected with OT (ICU/cell=50) for the indicated time periods. (B) LPS-primed BMDMs were infected with OT of the indicated ICU/cell for 6 h. (C) BMDMs were primed with LPS (10 ng/ml), live OT, heat-inactivated (HOT) or UV-inactivated (UVOT) for 16 h and then treated with ATP (5 mM) for 3 h. (D) LPS-primed BMDMs were challenged with ATP, live OT, heat-inactivated OT (HOT), or UV-inactivated OT (UVOT) for 6 h. (A-D) Error bars represent SD of triplicate samples. N.D.; not detected. (E) LPS-primed BMDMs were challenged with the vehicle (-), ATP (5 mM, for 3 h), *Salmonella enteritidis* (Sal., MOI=25) or OT (ICU/cell=50) for the indicated time periods. The caspase-1 activation was analyzed by western blotting using rabbit polyclonal antibodies specific for the p10 subunits of caspase-1. Data are representative of three independent experiments in A-E.

Since macrophages are a major source of IL-1β, we examined whether OT infection in macrophages *in vitro* can induce IL-1β release. LPS-primed BMDMs were infected with OT and levels of IL-1β release in the supernatant was assessed. As shown in Figure 2A and B, OT infection induced release of IL-1β in a time- and dose-dependent manner in LPS-primed BMDMs. The level of IL-1β secretion peaked 6 h postinfection and was proportional to the bacterial dose (ICU) (Figure 2A and B). Based on these results, we optimized the conditions for assessing IL-1β levels from OT-infected macrophages, and those conditions were used for further analysis.

In the current model of IL-1β maturation, two independent signals are required for production and secretion of active IL-1β [15]. The first signal is induced through plasma membrane-associated PRRs such as TLRs to activate transcription of pro-IL-1β, and the second signal is mediated by the NLR inflammasomes that induce cleavage of IL-1β precursors into the active forms through caspase-1 activation [31]. To determine whether OT provides the first or second signal, BMDMs were infected with live or inactivated bacteria in the presence or absence of priming with LPS. First, BMDMs were challenged with live, heat-inactivated or UV-inactivated OT for 16 h and then stimulated with ATP for IL-1β secretion. Although LPS-primed BMDMs released IL-1β, BMDMs pretreated with live or inactivated OT failed to effectively produce IL-1β upon ATP stimulation, indicating that OT, live or inactivated, provides a first signal at only modest levels to prime macrophages to synthesize pro-IL-1β (Figure 2C). Next, BMDMs were primed with LPS and challenged with ATP, or live or inactivated OT to determine whether OT infection provides a second signal for the processing and release of IL-1β. Live bacteria or ATP induced significant IL-1β release, but not heat- or UV-inactivated bacteria (Figure 2D). This suggests that only live OT was able to activate caspase-1 to induce IL-1β maturation and secretion. To confirm this, the OT-induced caspase-1 activation was assessed by western blot analysis. In addition to ATP-stimulated or *Salmonella*-infected BMDMs, the p10 fragment, a subunit of active caspase-1, was detected in BMDMs upon infection with live OT (Figure 2E). Taken together, these data suggest that OT activates caspase-1 and induces IL-1β secretion in macrophages.

### Phagocytosis of *O. tsutsugamushi* and Phagosomal Acidification were Required for IL-1β Release

**Figure 3:**
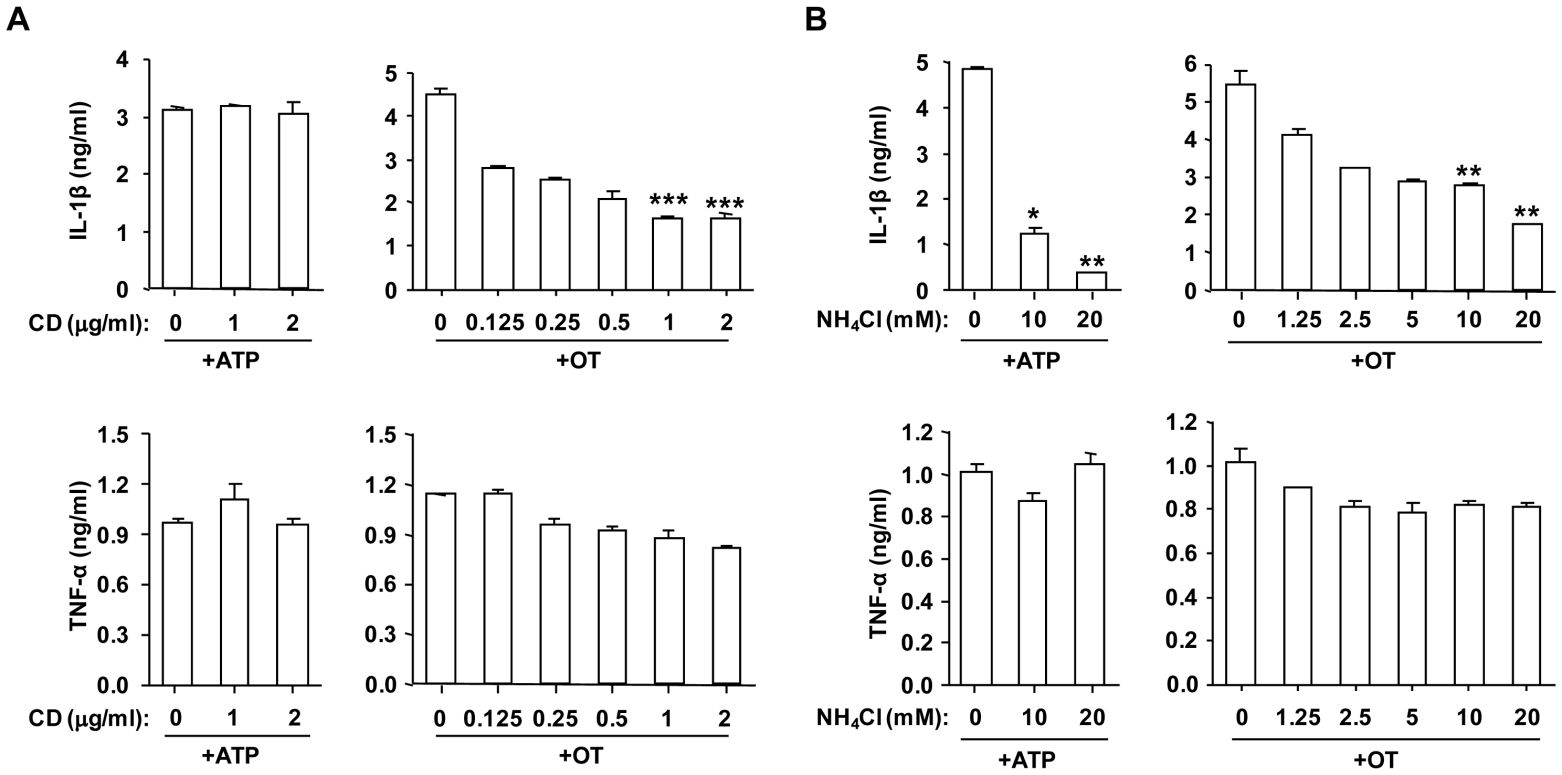
Bacterial internalization and endosomal acidification are required for efficient IL-1β secretion upon *O. tsutsugamushi* infection. LPS-primed BMDMs were pretreated with cytochalasin D (CD) (A) or NH_4_Cl (B) at the indicated concentration for 1 h, and then challenged with OT (50 ICU/cell) for 6 h or ATP (5 mM) for 3 h. The production of IL-1β and TNF-α from infected cells was assessed by ELISA. Error bars represent SD of triplicate samples. *p<0.05, **p<0.01, ***p<0.001 versus vehicle-treated cells. Data are representative of three independent experiments in A and B.

Since *O. tsutsugamushi* are obligatory intracellular bacteria, it is an interesting question to ask if intracellular invasion of OT is required for the activation of caspase-1. First, to determine whether phagocytosis is required for IL-1β secretion by BMDMs infected with OT, cells were pretreated for 1 h with various doses of actin polymerization inhibitor, cytochalasin D (CD), to inhibit phagocytosis. The CD treatment efficiently blocked *Orientia* internalization (data not shown). CD pretreatment reduced mature IL-1β release from LPS-primed BMDMs infected with OT in a dose-dependent manner but did not affect ATP-induced IL-1β release (Figure 3A). On the other hand, TNF-α release in response to live OT was not affected by CD pretreatment. These results further demonstrate that live intracellular *Orientia* organisms are required to process IL-1β. Next, to examine if phagosomal acidification is important for OT-induced IL-1β secretion, ammonium chloride, a chemical agent that blocks endosome acidification, was added to the culture. NH_4_Cl treatment reduced IL-1β release from both OT infected and ATP treated macrophages in a dose-dependent manner, indicating that endo/phagosomal maturation is required for IL-1β secretion upon OT infection (Figure 3B). There was no significant change in TNF-α levels upon NH_4_Cl treatment. Therefore, these data demonstrate that IL-1β processing upon OT infection requires active uptake of living bacteria and maturation of phagosomes.

### Caspase Activation is Essential for IL-1β Release from OT-infected Macrophages

**Figure 4:**
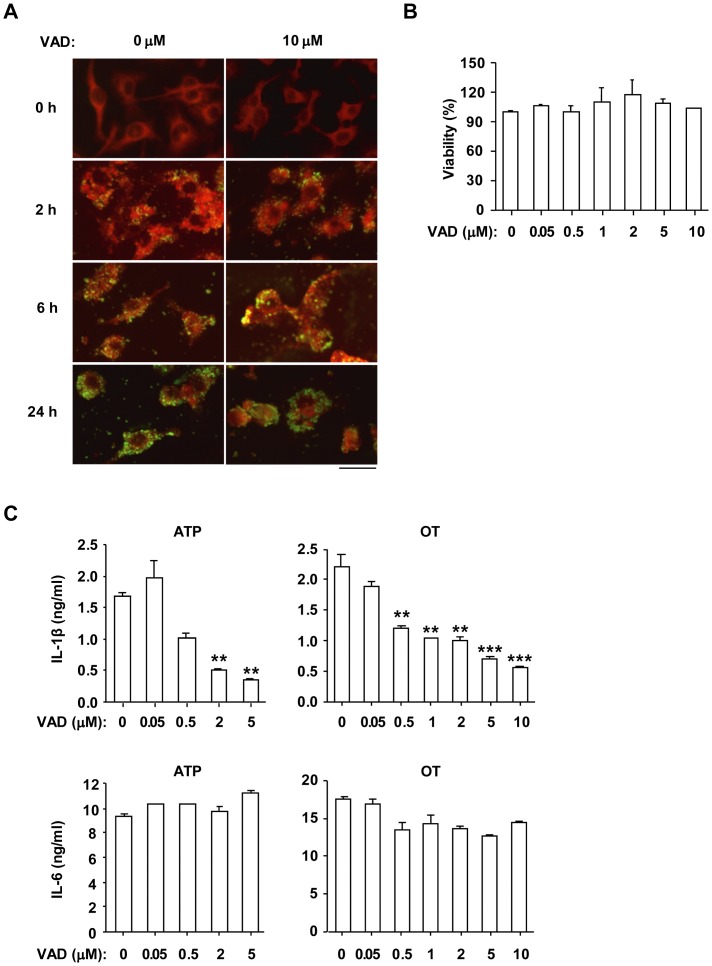
IL-1β secretion induced by *O. tsutsugamushi* infection in LPS-primed BMDMs was blocked by pan-caspase inhibitor. LPS-primed BMDMs were pretreated with pan-caspase inhibitor Z-VAD-FMK (VAD) at the indicated concentration for 1 h and then challenged with ATP (5 mM) for 3 h or OT (A. ICU/cell=10; B,C. ICU/cell=50) for the indicated period (A) or 6 h (B, C). (A) Cells were fixed and stained with human antiserum against OT and a FITC-conjugated goat anti-human IgG antibody, and examined using a fluorescence microscope. Green spots indicate OT and red areas indicate the host cell. Scale bar, 50 µm. (B) Cell viability was measured by MTT assay. (C) The production of IL-1β and IL-6 was assessed by ELISA. Error bars represent SD of triplicate samples. **p<0.01, ***p<0.001 versus vehicle-treated cells. Data are representative of three independent experiments in A-C.

To investigate whether OT-infected BMDMs require caspase activation for release of IL-1β, the pan-caspase inhibitor, Z-VAD-FMK was used. First, we performed immunofluorescence microscopy and MTT assay in order to test whether Z-VAD-FMK has any inhibitory effect on bacterial internalization into host cells and host cell viability. The LPS-primed BMDMs were pretreated with various doses of Z-VAD-FMK, and then infected with OT. Z-VAD-FMK did not interfere with internalization of OT into the cells (Figure 4A). Furthermore, the indicated concentration of inhibitor did not affect the viability of BMDMs infected with OT (Figure 4B). LPS-primed BMDMs were pretreated with Z-VAD-FMK in various concentrations, and then infected with OT or treated with ATP. The level of IL-1β in the culture supernatant was significantly reduced by treatment with the pan-caspase inhibitor (Figure 4C). On the other hand, the caspase inhibitor had no significant effect on the level of IL-6 (Figure 4C). These results suggest that activation of caspase is essential for OT-induced IL-1β secretion by BMDMs.

### *O. tsutsugamushi* Infection does not Induce Pyroptotic Cell Death in Bone Marrow-Derived Macrophages

**Figure 5:**
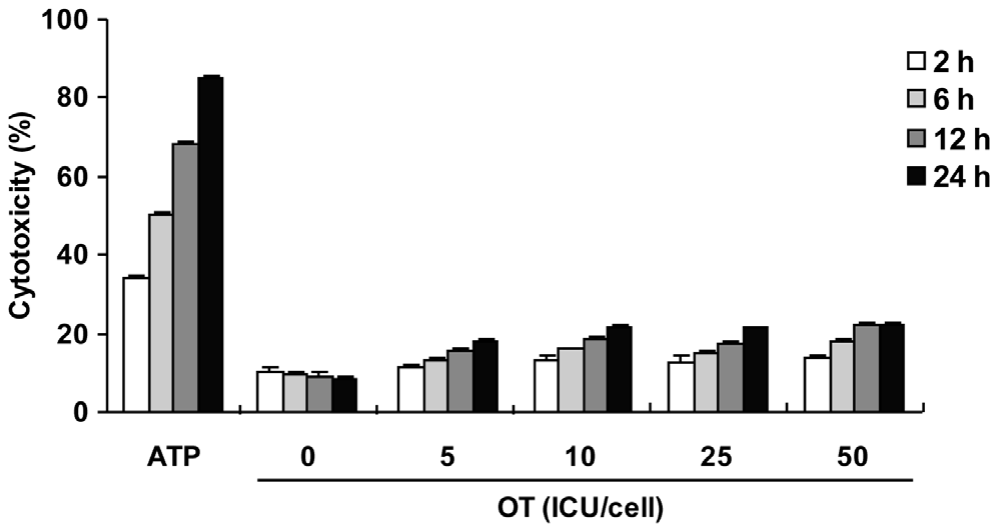
*O. tsutsugamushi* infection did not induce pyroptosis. LPS-primed BMDMs were challenged with ATP (5 mM) or OT with the indicated ICU/cell for the indicated time periods. Pyroptosis was assessed by LDH release. Error bars represent SD of triplicate samples. Data are representative of three independent experiments.

Pyroptosis is a caspase-1-dependent cell death process through ASC pyroptosome, a molecular platform to recruit and activate caspase-1, largely composed of oligomerized ASC [32]. Pyroptosis is an important protection mechanism of the host by eliminating infected cells, and various intracellular bacterial species such as *Salmonella* or *Legionella* have been shown to induce pyroptosis via caspase-1 activation [33], [34]. To determine whether *O. tsutsugamushi* infection can induce pyroptosis, LPS-primed macrophages were infected with various doses of bacterium for the indicated time periods, and then culture supernatants were used for lactate dehydrogenase (LDH) release assay. The ATP treatment induced significant release of LDH into the culture medium as previously described [35], [36]. However, *O. tsutsugamushi* infection did not induce pyroptosis even at high doses of bacterium in wild-type macrophages as well as *Nlrp3*-, *Nlrc4*-, *Aim2*- and *Asc*-deficient macrophages (Figure 5 and data not shown). Therefore, *O. tsutsugamushi* infection does not induce pyroptosis, although it effectively induces the inflammasome to activate caspase-1.

### Nlrp3 and Nlrc4 do not Play a Major Role in the Production of IL-1β Induced by *O. tsutsugamushi* Infection

**Figure 6:**
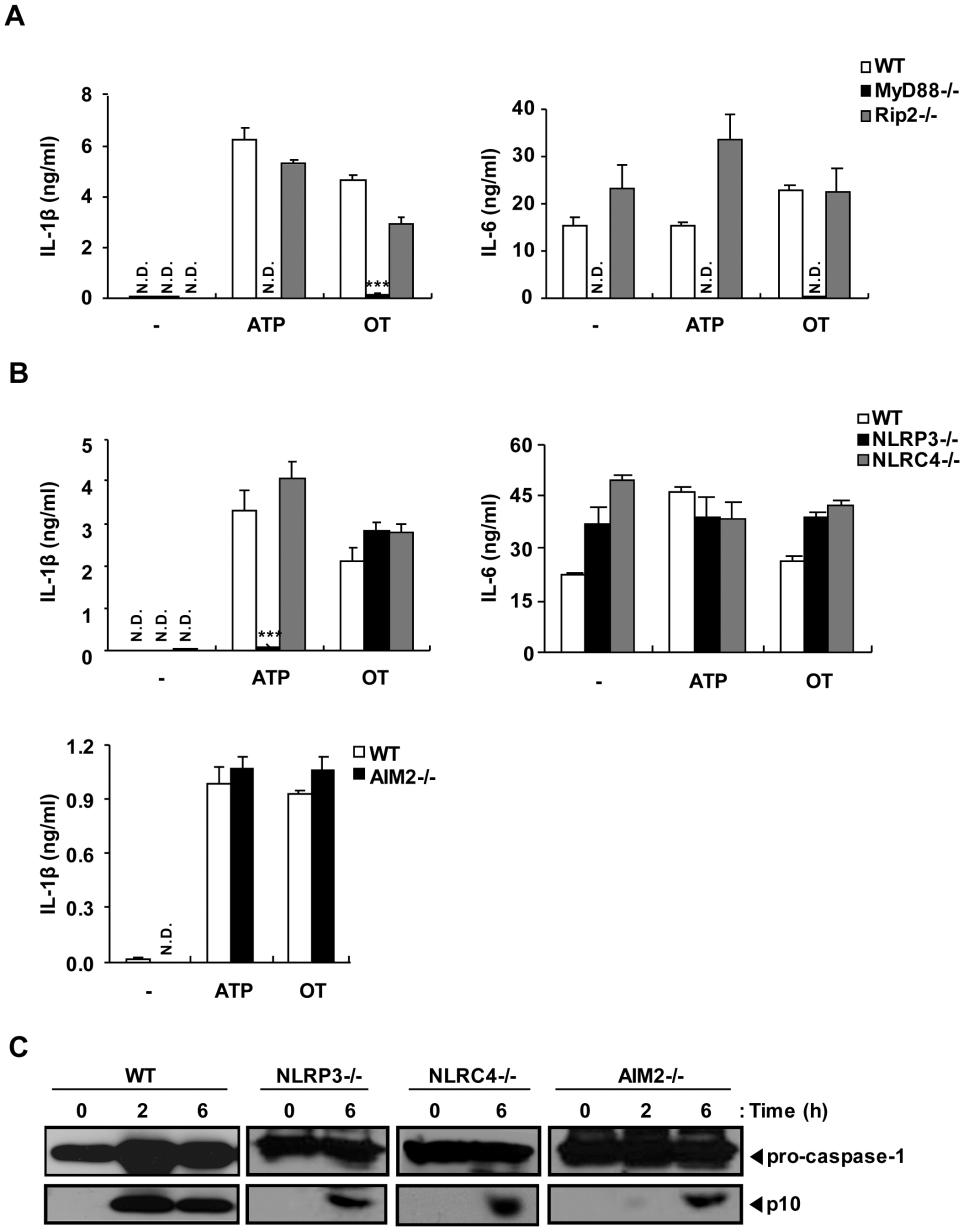
*O. tsutsugamushi* activates caspase-1 in LPS-primed BMDMs in Nlrp3-, Nlrc4- and AIM2-independent manners. Wild-type (WT), *MyD88*-, *Rip2*-, *Nlrp3*-, *Nlrc4*- or *Aim2*-deficient BMDMs were primed with LPS (10 ng/ml) for 16 h and then treated with ATP (5 mM) for 3 h or infected with OT (ICU/cell=50) for 6 h or the indicated time periods. (A, B) IL-1β and IL-6 production from the cells was assessed by ELISA. (C) The cleaved caspase-1 and procaspase-1 were analyzed by western blotting using rabbit polyclonal antibodies specific for the p10 subunits of caspase-1. Error bars represent SD of triplicate samples. N.D.; not detected. -; untreated. ***p<0.001 versus wild-type. Data are representative of three independent experiments in A-C.

The caspase-1 activation induced by *O. tsutsugamushi* infection suggested the possible involvement of inflammasome-forming NLRs in the detection of intracellular OT. To gain insight into the signals activated by the internalization of OT, macrophages from wild-type and mutant mice were infected with OT. To investigate whether caspase-1 activation by OT was dependent on TLR or Nod1/Nod2 signaling, *MyD88*- and *Rip2*-deficient BMDMs were primed by LPS treatment, and then infected with OT or treated with ATP. IL-1β release upon ATP stimulation was not detectable in *MyD88*-deficient BMDMs but was not altered in *Rip2*-deficient BMDMs. These results were consistent with the previous reports, since TLR4 signaling via the MyD88-dependent pathway is required as a first signal for pro-IL-1β synthesis upon LPS priming (Figure 6A). Similarly, upon OT infection, wild-type and *Rip2*-deficient BMDMs produced both IL-1β and IL-6 significantly, whereas *MyD88*-deficient BMDMs did not. These suggest that MyD88-dependent signaling is required for LPS priming whereas Rip2 is not required for LPS priming and inflammasome formation in OT*-*infected macrophages. To determine which NLR or HIN-200 family proteins are involved in OT induced inflammasome activation, the LPS-primed macrophages from wild-type, *Nlrp3*-, *Nlrc4*-, or *Aim2*-deficient mice were challenged with OT or ATP, and the production of IL-1β was assessed by ELISA. The production of IL-1β induced by ATP treatment was dependent on Nlrp3, as previously shown (Figure 6B) [37], [38]. However, *Nlrp3*-, *Nlrc4*-, or *Aim2*-deficient macrophages infected with OT produced IL-1β at a level comparable to that of wild-type macrophages (Figure 6B). All types of macrophages produced the inflammasome-independent proinflammatory cytokine IL-6 in response to OT infection.

Secretion of biologically active IL-1β requires posttranslational processing by active caspase-1. Therefore, we compared caspase-1 activation in wild-type and *Nlrp3*-, *Nlrc4*-, or *Aim2*-deficient BMDMs by western blotting for p10 caspase-1 subunits that are generated by autocatalytic cleavage and released from the cell by a poorly defined mechanism [39]. Culture supernatants from LPS-primed wild-type BMDMs contained the caspase-1 p10 subunit after infection with OT (Figure 6C). LPS-primed BMDMs from *Nlrp3*-, *Nlrc4*-, or *Aim2*-deficient mice were also able to process caspase-1 to produce the caspase-1 p10 subunit in response to OT (Figure 6C). These data indicate that Nlrp3, Nlrc4 and AIM2 do not play significant roles in the caspase-1 activation during OT infection.

### Caspase-1 and ASC are Essential for *O. tsutsugamushi*-induced IL-1β Secretion in Bone Marrow-derived Macrophages

**Figure 7:**
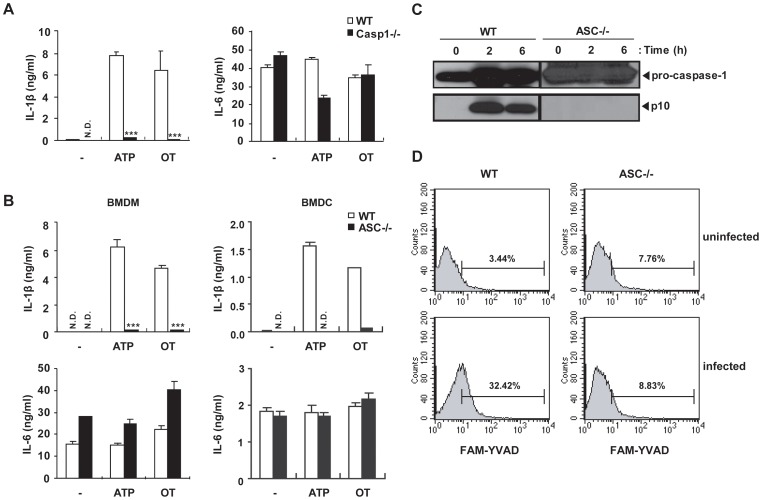
Live *O. tsutsugamushi* activates caspase-1 in LPS-primed BMDMs in caspase-1- and ASC-dependent manners. (A) Wild-type (WT) or *caspase-1*-deficient BMDMs were primed with LPS (10 ng/ml) for 16 h and then treated with ATP (5 mM) for 3 h or infected with OT (ICU/cell=50) for 6 h. IL-1β and IL-6 production from infected cells was assessed by ELISA. (B) Wild-type (WT) or *Asc*-deficient BMDMs (left panel) or BMDCs (right panel) were primed with LPS and treated as in panel A. IL-1β and IL-6 production from infected cells was assessed by ELISA. (C) Wild-type (WT) or *Asc*-deficient BMDMs were primed with LPS (10 ng/ml) for 16 h and then infected with OT (ICU/cell=50) for the indicated time periods. The cleaved caspase-1 and procaspase-1 were analyzed by western blotting using rabbit polyclonal antibodies specific for the p10 subunits of caspase-1. (D) Caspase-1 activation in LPS-primed wild-type (WT) or *Asc*-deficient BMDMs after infection with *O. tsutsugamushi*. Numbers above bracketed lines indicate the percent of cells positive for FAM–YVAD staining. Error bars represent SD of triplicate samples. N.D.; not detected. ***p<0.001 versus wild-type. Data are representative of three independent experiments in A-D.

The activation of pro-caspase-1 is caused by inflammasome activation either in an adaptor ASC-dependent or –independent manner [40]. To investigate whether ASC is required for IL-1β production by OT infection, macrophages from caspase-1-deficient mice and *Asc*-deficient mice were primed with LPS and then challenged with OT or ATP. Caspase-1-deficient macrophages failed to produce IL-1β (Figure 7A). Similar to caspase-1-deficient macrophages, *Asc*-deficient macrophages, as well as dendritic cells, did not produce IL-1β (Figure 7B). Furthermore, *Asc*-deficient macrophages were not able to process caspase-1 upon OT infection, evidenced by the absence of active caspase-1 by western blotting analysis and flow cytometric analysis using an active caspase-1-specific fluorescent probe (FAM-YVAD-FMK) (Figure 7C and D). These data indicate that caspase-1 is essential for secretion of IL-1β in response to cytosolic *Orientia* and identify ASC as the critical inflammasome adaptor for caspase-1 activation in response to OT infection.

## Discussion

**Figure 8:**
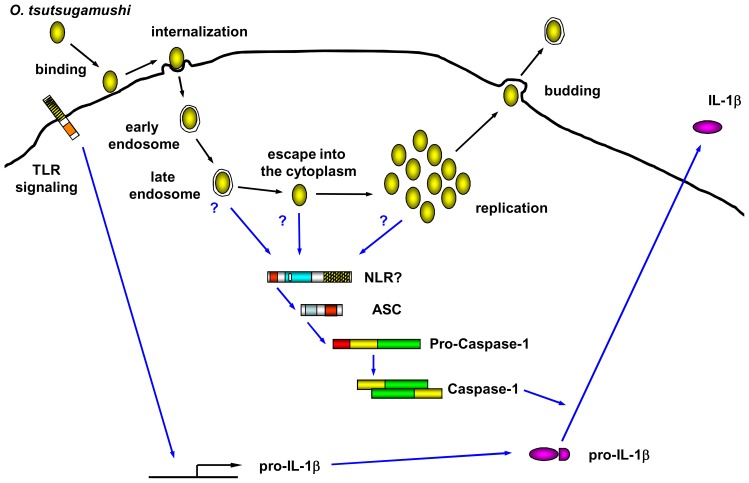
Model of inflammasome activation by *O. tsutsugamushi*. OT infection requires attachment to the host cell surface (binding), followed by uptake of the bacteria by clathrin-mediated endo/phagocytosis (internalization). After maturation of the endo/phagosome, OT can be released into the cytoplasm (escape), where OT multiplies (replication). OT in the mature endo/phagosome or cytoplasm activates ASC inflammasome, which induce activation of caspase-1. Active caspase-1 processes pro-IL-1β cleavage, which results in the maturation and secretion of IL-1β.

The innate immune cells recognize pathogenic microbial invasion and produce proinflammatory cytokines such as IL-1 and TNF-α to activate the immune system and cause inflammation. In this study, we found that OT infection induces ASC inflammasome activation and IL-1β secretion which is a key innate immune response against bacterial invasion. Indeed, IL-1 receptor signaling is important for the host defense since IL-1R deficient mice were susceptible to OT infection. Interestingly, live OT but not inactivated OT induces caspase-1 activation and IL-1β secretion in LPS-primed BMDMs (Figure 2). The OT exploits integrin-mediated signaling and the actin cytoskeleton for internalization of host cells (Figure 8) [41]. The actin polymerization inhibitor effectively blocked IL-1β processing and release by OT infection (Figure 3A). Following internalization, the phagosome is transformed into a phagolysosome through a progressive maturation process that is dependent on the sequential fusion of lysosomes with the internalized phagosome (Figure 8) [42], [43]. After internalization into the host cell by endo/phagocytosis, OT escapes from the late endo/phagolysome to the cytosol (Figure 8) [44]. Although the mechanism behind this OT escape from the endo/phagolysome is not clear, it has been shown that OT escapes from the endosome and the infectivity of OT is impaired in the presence of inhibitors for endosomal maturation such as NH_4_Cl or bafilomycin. Thus, the acidification of endocytic compartments is required for the efficient OT infection [44]. Interestingly, endosomal acidification inhibitor-treated BMDMs reduced release of IL-1β by *Orientia* infection (Figure 3B). Therefore, OT internalization and phagosomal acidification are required for IL-1β release by BMDMs. These findings suggest that OT*-*induced IL-1β secretion requires bacterial escape from the phagosome to the cytosol where the bacterium replicates and the PAMPs of OT may activate one of NLRs in the cytoplasm (Figure 8). In case of infection of *Ehrlichia muris*, another obligate intracellular bacterium in the family of *Anaplasmataceae,* BMDMs do not induce IL-1β secretion, probably because *E. muris* resides in endosomal compartments and does not escape into the cytosol [45]. Another possible mechanism behind how OT activates cytoplasmic innate immunity might be due to the secretion of active PAMPs by a type IV secretion system [46]. In this case, however, it is not clear why acidification of phagosomes is required for caspase-1 activation.

IL-1β secretion from OT-infected BMDMs indicates that component(s) of the inflammasome may recognize this bacterium in the cytosol. Although *Asc*- or caspase-1-deficient BMDMs did not induce IL-1β secretion, the OT infection-induced IL-1β secretion and caspase-1 activation were not affected by *Nlrp3*-, *Nlrc4*-, or *Aim2*-deficiency (Figure 6). ASC is an adaptor protein which interbridges between Pyrin domain containing cytoplamic sensor and CARD domain containing caspase-1. Therefore, it is likely that Pyrin containing NLR may play an important role in cytosolic detection of OT. Our data indicate that Nlrp3 is unlikely to be the candidate (Figure 6). Thus, it is unlikely that Nlrp3-activating factors such as potassium efflux, generation of reactive oxygen species or cytoplasmic release of lysosomal enzymes are associated with OT-induced inflammasome activation. Also, we have not seen the reduction of IL-1β production in *Nlrp6-* and *Nlrp12*-deficient macrophages (data not shown). Since macrophages are major producer of IL-1β, these data suggests that Nlrp3, Nlrp6, Nlrp12, Nlrc4 and Aim2 are unlikely to be involved in OT-induced inflammasome activation. In human, 14 out of 23 NLRs are Pyrin containing NLR (NLRP) and more exists in mouse. Further analysis is required for the identification of OT-sensing NLR in the cytoplasm.

Although live OT can activate inflammasome, OT, whether live or not, were not very effective in priming macrophages for subsequent stimulation for IL-1β production (Figure 2C). We found that *in vivo* infection of OT induced IL-1β in the serum, suggesting that OT is able to do both priming and inflammasome activation during *in vivo* infection. Perhaps OT can prime cells more efficiently via TLR signaling *in vivo* than *in vitro* in BMDMs, and this results in the IL-1β production in OT infected animals. Another interesting possibility is that priming of cells during *in vivo* OT infection may be enhanced by cytokine signaling through positive feedback loop via the production of proinflammatory cytokines such as TNF-α.

It has been demonstrated that caspase-1 activation also leads to cell death called pyroptosis during microbial infection [32]. Pyroptosis mediates further activation of inflammatory response by the innate immune system. Upon infection by intracellular bacteria or viruses, infected cells form an ASC pyroptosome, which rapidly recruits and activates caspase-1 resulting in pyroptosis and the release of the intracellular proinflammatory cytokines [32]. *Francicella tulerensis*, for instance, activates AIM2 inflammasome leading to IL-1β processing and pyroptosis through ASC pyroptosome formation [29]. *Legionella* spp induce pyroptosis via Nlrc4/Birc1e inflammasome in general, however, *Legionella* strains which do not induce pyroptosis, sush as *L. parisiensis* or *L. tucsonensis* or flagellin-defective *L. pneumophila*, can replicate efficiently in macrophages, indicating critical roles of pyroptosis for host defense [47]. Interestingly, although OT infection activates caspase-1, OT-infected macrophages did not release the LDH significantly, which is different from other bacteria that induce pyroptosis (Figure 5). *Nlrp3-, Nlrc4-, Aim2-, Asc-* and caspase-1-deficient macrophages also did not undergo pyroptosis upon *Orientia* infection (data not shown). Our results suggest that *Orientia* perhaps actively inhibits pyroptosis of infected macrophages, resulting in better survival of host cells and poorer eradication of OT from infected cells and tissues. It has also been reported that OT can inhibit beauvericin-induced apoptosis of THP-1 cells, by modulating intracellular mobilization of Ca_2_^+^, which may account for the anti-apoptotic function of OT in macrophages [48]. Regulation of viability of host cells might play a significant role in efficient infectivity and proliferation of OT.

Although we have not identified the NLR that is responsible for OT recognition, we suggest the possibility that cytosolic PRR is able to recognize OT and lead to innate immune response by secreting IL-1β. Interestingly, OT can survive in the host cell, presumably by disturbing the host cell death mechanism. Further study is needed to define the NLR inflammasome and ligand for IL-1β secretion upon OT infection, and to identify the mechanism which regulates pyroptosis.

## Materials and Methods

### Bacteria and *in vitro* Orientia tsutsugamushi Infection of Cells

The prototype strain, *Orientia tsutsugamushi* Boryong was propagated in monolayer of L-929 cells (American Type Culture Collection) as described previously [49]. When more than 90% of the cells were infected, the cells were collected, homogenized with a glass Dounce homogenizer (Wheaton Inc., NJ, USA), and centrifuged at 500× *g* for 5 min at 4°C. The supernatant was recovered and stored in liquid nitrogen until use. The infectivity titer of the inoculum was determined as described previously [49]. Briefly, five-fold serially diluted oriential samples were inoculated onto L-929 cell layers on 24-well tissue culture plates. After 3 days of incubation, the cells were collected, fixed, and stained using an anti-orientia antibody. The ratio of infected cells to the counted number of cells was determined microscopically, and infected-cell counting units (ICU) of the oriential sample were calculated as follows: ICU = (total number of cells used in infection) × (percentage of infected cells) × (dilution rate of the orientiae suspension)/100. Heat-inactivation of bacterial inoculum was performed by heating OT at 56°C for 30 min. UV-inactivated bacterial inoculum was obtained by exposure to 254 nm wavelength UV of 4 mW⋅ sec/cm^2^ for 30 min.

In the inhibition assays, BMDMs were primed with LPS (from *Salmonella minnesota,* Alexis, NY, USA) for 16 h, then were preincubated with cytochalasin D (CD; Sigma, MO, USA), ammonium chloride (NH_4_Cl, Sigma), or Z-VAD-FMK (Calbiochem, NJ, USA). for 1 h before OT was inoculated. Inhibitors were maintained for the course of inhibition assays. After 1 h incubation, cells were infected with OT for the indicated time periods. Cell viability was determined by 3-(4,5-dimethyl-2,5 thiazolyl)-2,5 diphenyl tetrazolium bromide (MTT) assay and was not affected by doses of the inhibitors used in this study.

### Mice

*MyD88*-, *Rip2*-, *caspase-1*-, *ASC*-, *Nlrc4*-, *Nlrp3*-, and *Aim2*-deficient mice were established as described, and the mice or femurs were kindly provided by Drs. Shizuo Akira (Osaka University), Richard Flavell, Yasunori Ogura (both at Yale University), Vishva Dixit, Sanjeev Mariathasan (both at Genentech), Katherine Fitzgerald and Vijay Rathinam (both at University of Massachusetts) [22], [26], [37], [50], [51], [52]. C57BL/6 mice were from Taconic Farm, Inc and Orient Bio Inc. (Gyeonggi-do, South Korea). *IL-1R*-deficient mice were originally from the Jackson Laboratory [53]. Mice were maintained under specific pathogen-free conditions. All mice were maintained and used in accordance with institutional and National Institutes of Health guidelines. All animal procedures were approved by and performed according to the guidelines of the Institutional Animal Care and Use Committee of Dana-Farber Cancer Institute (#04-044 and 04-045) and/or Jeju National University (#2010-0028).

### *Orientia* Challenge of Mice *in vivo*

The female C57BL/6 mice were infected by intraperitoneal (*i.p.*) injection of 0.1 ml of oriential suspension (5×10^6^ ICU). On the indicated days of infection, mice were sacrificed, and whole blood and spleens were obtained.

### RNase Protection Assay

Total RNA was extracted from the spleen using Trizol Reagent (Gibco BRL, Grand Island, NY) and was quantitated spectrophotometrically. Detection and semiquantitation of various murine cytokine and chemokine mRNAs were accomplished with the RiboQuant multiprobe RNase protection assay kit from PharMingen (San Diego, CA). In brief, a mixture of [^32^P]UTP-labeled antisense riboprobes was generated from a panel of different cytokine or chemokine template DNAs. These panels also included templates for the murine housekeeping genes encoding GAPDH and L32 to ensure equal loading of total RNA onto the gels. Total RNAs from each sample (30 µg each) were hybridized overnight at 56°C with 3×10^6^ cpm of the ^32^P-labeled antisense riboprobe mixture. After hybridization, the samples were digested with a mixture of RNases A and T_1_. Nuclease-protected RNA fragments were precipitated with ethanol. The samples were resolved on a 5% polyacrylamide sequencing gel. The bands were observed after autoradiography. The specific cytokine and chemokine bands were identified on the basis of their individual mobilities compared with the undigested probes.

### Determination of Bacterial Load in Tissues

Since *O. tsutsugamushi* is an obligatory intracellular bacterium that does not grow outside of the cells, quantification method for the *Orientia* load using qPCR was used with a modification of previously established methods by other groups [54], [55]. Briefly, the oriential load was determined by qPCR (with SYBR Green) for the *Orientia tsa56* gene, which encodes a 56-kDa type-specific antigen of *O. tsutsugamushi*. Primer sequences are as follow: *O. tsutsugamushi tsa56* forward, AACCCTAATCCTGTTGGACAGCCA; *O. tsutsugamushi tsa56* reverse, ACTTTGACAGGAGAAGCGCTAGGT; mouse GAPDH forward, CAACTACATGGTCTACATGTTC; and GAPDH reverse, CTCGCTCCTGGAAGATG. The substrate for amplification was DNA purified from samples using the DNeasy Tissue kit (Qiagen, Valencia, CA). qPCR was performed using the 7300 real time PCR system from Applied Biosystems. Results were normalized to GAPDH levels in the same sample and expressed as relative *Orientia tsa* amounts per GAPDH amount.

### Bone Marrow-derived Macrophages (BMDMs)

The bone marrow cells from wild-type and mutant mice were obtained from tibia and femur of mice by flushing with DMEM (Invitrogen, CA, USA) containing 10% heat-inactivated FBS, 100 U of penicillin G and streptomycin. The 1×10^7^ bone marrow cells were cultured in 10 ml of DMEM medium containing glutamine, 20% heat-inactivated FBS, 100 U of penicillin G, streptomycin and 30% L929 cell supernatant containing M-CSF in 100 mm petri dish (BD Falcon, NJ, USA) at 37°C in humidified 5% CO_2_ for 6 days. At day 6 of culture, cells were harvested with cold PBS, washed, resuspended in DMEM supplemented with 10% FBS and used at a density of 2×10^5^ cells/well in a 24 well plate for experiments.

### Bone Marrow-derived Dendritic Cells (BMDCs)

DCs were grown from wild-type and various knockout mice. Briefly, bone marrow from tibia and femur was obtained as described above, and bone marrow cells were cultured in RPMI 1640 medium containing 10% heat-inactivated FBS, 50 µM of 2-ME, and 2 mM of glutamine supplemented with 3% J558L hybridoma cell culture supernatant containing GM-CSF. The culture medium containing GM-CSF was replaced every other day. At day 6 of culture, nonadherent cells and loosely adherent DC aggregates were harvested, washed, resuspended in RPMI 1640 supplemented with 5% FBS and used at a density of 2×10^5^ cells/ml for experiments unless mentioned otherwise.

### LDH Release Assay

BMDMs were dispensed to 48-well culture plates at a concentration of 1×10^5^/0.5 ml, and incubated for 24 h at 37°C in humidified 5% CO_2_. Cells were primed with LPS (10 ng/ml), and then further incubated for 16 h. LPS-primed BMDMs were treated with ATP or infected with the indicated ICU of OT. After 1 h, the infected cells were washed, and then incubated for the indicated time periods. Cell death was quantified with the CytoTox96 LDH-release kit (Promega, WI, USA). Percent of cell death is calculated measuring the OD490 of each sample and using the following formula: [(experimental cells-untreated cells)/(lysed cells-untreated cells)] ×100%.

### Indirect Immunofluorescent Antibody (IFA) Test

BMDMs were dispensed to Lab-Tek chamber slides (Nunc, NY, USA) at a concentration of 4×10^4^/0.2 ml, and incubated for 24 h at 37°C in humidified 5% CO_2_. Cells were primed with LPS (10 ng/ml), then further incubated for 16 h. LPS-primed BMDMs were treated with several doses of inhibitor for 1 h, and then infected with OT. Slides were removed from the Lab-Tek chamber, then cells were fixed with acetone. The slide was incubated with diluted human antiserum against OT for 30 min at 37°C and washed three times with PBS for 5 min by shaking. After the slide was dried, it was stained with goat anti-human IgG-FITC conjugates (Caltag, CA, USA) and incubated in the dark at 37°C for 30 min. The slide was washed three times with PBS for 5 min by shaking. The counter-staining was performed with 0.003% Evans Blue (Sigma) solution in PBS for 1 min, and then washed two times in PBS for 1 min by shaking. Finally, the cover slip was mounted using mounting media (90% glycerol, 10% PBS). The stained cells were examined with a fluorescence microscope.

### Western Blot Analysis

Bone marrow-derived macrophages (BMDMs) were dispensed to 35 mm culture dishes at a concentration of 2×10^6^ cells/2 ml and cultured for 24 h, and then cells were primed with LPS (10 ng/ml) for 16 h and treated with ATP or infected with OT for the indicated time periods. The culture supernatants were collected and precipitated by addition of an equal volume of methanol and 0.25 volumes of chloroform. The supernatant/methanol/chloroform mixtures were vortexed and then centrifuged for 10 min at 20,000× *g*. The upper phase was discarded and 500 µl of methanol was added to the interphase. This mixture was centrifuged for 10 min at 20,000× *g* and the protein pellet was dried, resuspended in protein sample buffer and boiled for 5 min. Protein concentration in each sample was determined using a bicinchoninic acid protein assay kit (Sigma). Protein samples were electrophoresed in 15% SDS-polyacrylamide gels and transferred to a polyvinylidene fluoride (PVDF) membrane (Bio-Rad, CA, USA). The membrane was incubated overnight with 1/250-diluted rabbit polyclonal antibodies specific for the p10 subunits of caspase-1 (Santa Cruz Biotechnology, CA, USA) in blocking buffer at 4°C. After washing, the membrane was incubated with a horseradish peroxidase (HRP)-linked anti-rabbit IgG (Cell Signaling, MA, USA) as a secondary antibody. Immunoactive bands were detected using the WEST-ZOL plus Western blot detection system (iNtRON Biotechnology, Seoul, South Korea) according to the manufacturer’s instructions.

### Flow Cytometry for Active Caspase-1 Staining

Active caspase-1 staining was assessed by flow cytometry. LPS-primed bone marrow-derived macrophages (1×10^6^ BMDMs) in non-tissue-culture-treated 12-well plates were infected for 4 h with *O. tsutsugamushi* (ICU/cell=5). Cells were removed with cold PBS and stained for 1 h with FAM–YVAD–fluoromethylketone ((FAM–YVAD–FMK; Immunochemistry Technologies, MN) as recommended by the manufacturer. Data were acquired on a FACSCalibur (Becton Dickinson) and were analyzed with CellQuest software (Becton Dickinson).

### Cytokine Measurement

2×10^5^ BMDMs were seeded onto 24-well plate, and then incubated for 24 h at 37°C in humidified 5% CO_2_. After treatment with LPS (10 ng/ml) for 16 h, macrophages were infected with OT (ICU/cell=50) for 6 h or ATP (5 mM) for 3 h. Culture supernatants were collected from culture plates and were centrifuged at 14,000× *g* for 5 min at 4°C. Concentrations of murine IL-1β (R&D system, MN, USA), IL-6 (BD PharMingen, CA, USA), and TNF- α (R&D system) in the culture supernatants or in the mice serum were determined by enzyme-linked immunosorbent assay (ELISA) according to the manufacturer’s instructions.

### Statistical Analysis

All experiments were performed at least three times. Data are expressed as mean ± standard deviation (SD). Analysis of variance (ANOVA) was used to evaluate the data with the following significance levels: *p<0.05, **p<0.01, ***p<0.001.
